# From Cheese Whey to Functional Ingredients: Upcycling Whey Proteins for Cardiovascular and Immunomodulatory Health—Evidence Mapping and Perspectives from Portugal

**DOI:** 10.3390/foods15050908

**Published:** 2026-03-06

**Authors:** João Mota, Márcio Moura-Alves, Ana Francisca Teixeira, Rafaela Nóbrega, Diogo Lameirão, Carla Gonçalves

**Affiliations:** 1 LAQV-REQUIMTE, Department of Chemistry, University of Aveiro, Campus Universitário Santiago, 3810-193 Aveiro, Portugal; joao.mota170@gmail.com; 2 CECAV—Centre for Studies in Animal and Veterinary Science, University of Trás-os-Montes and Alto Douro (UTAD), 5000-801 Vila Real, Portugal; mmalves@utad.pt; 3 AL4AnimalS—Associate Laboratory for Animal and Veterinary Sciences, 1300-477 Lisboa, Portugal; 4 Biology and Environment Department, University of Trás-os-Montes and Alto Douro (UTAD), 5000-801 Vila Real, Portugal; anafrancisca033@gmail.com (A.F.T.); rafaelanobrega.nutricao@gmail.com (R.N.); diogolameirao@gmail.com (D.L.); 5 CITAB—Centre for the Research and Technology of Agro-Environmental and Biological Sciences, University of Trás-os-Montes and Alto Douro (UTAD), 5000-801 Vila Real, Portugal; 6 RISE-Health, School of Life and Environmental Sciences, University of Trás-os-Montes and Alto Douro (UTAD), 5000-801 Vila Real, Portugal

**Keywords:** cheese whey, whey proteins, bioactive peptides, circular economy, immunomodulatory effects, dairy by-product valorization, functional ingredients

## Abstract

Cheese whey, a low-value by-product of cheese production, has gained renewed attention within the transition toward sustainable and circular food systems. Despite posing environmental challenges due to its high biochemical and chemical oxygen demand, whey retains a substantial proportion of milk nutrients, notably high-quality proteins that can be converted into bioactive peptides with potential health benefits. These peptides have been shown to modulate key biological pathways, including angiotensin-converting enzyme inhibition, nitric oxide bioavailability, oxidative stress balance, and inflammatory signaling, providing mechanistic plausibility for cardioprotective and immunomodulatory effects. However, the translation of promising in vitro and animal findings into consistent human health outcomes remains constrained by variability in peptide composition, processing conditions, bioavailability, and study design. This narrative review critically synthesizes current evidence on the functional properties of whey-derived peptides, with particular emphasis on cardiovascular and immunomodulatory outcomes across experimental models. In addition, the review situates whey upcycling within the Portuguese agro-food context, highlighting regional cheese production as both an environmental challenge and an opportunity for sustainable innovation. By integrating mechanistic evidence with sustainability-driven valorization strategies, this review aims to clarify the translational potential of whey-derived peptides as functional food ingredients.

## 1. Introduction

Cheese whey, long regarded as a low-value by-product of dairy processing, has renewed attention within the transition toward sustainable and circular food systems [1,2,3,4]. Although its disposal poses environmental challenges due to high biochemical oxygen demand, whey retains a substantial fraction of milk nutrients, including high-quality proteins and bioactive peptides that can be transformed into value-added ingredients [3,5,6,7]. In parallel with global efforts focused on resource efficiency and waste reduction, interest in whey upcycling has accelerated, driven by the convergence of environmental sustainability, functional food innovation, and advances in protein and peptide research [5,6,8].

A particularly promising direction in whey upcycling is the generation of bioactive peptides that could support cardiovascular and immune health [9,10]. Advances in proteomics, enzymatic hydrolysis, fermentation, and membrane-based separation have enabled the identification and characterization of diverse peptide sequences released from whey proteins [11,12]. Many of these peptides interact with biological pathways central to vascular and immune regulation, including angiotensin-converting enzyme (ACE) activity, nitric oxide (NO) signaling, oxidative balance, and inflammatory modulation, providing mechanistic plausibility for cardioprotective and immunomodulatory effects [9,13,14]. Despite encouraging mechanistic and preclinical evidence, translating laboratory observations into consistent human benefits remains challenging. Variability in peptide composition, limited standardization across studies, and the scarcity of long-term clinical trials continue to constrain the incorporation of whey-derived peptides into validated functional food applications [12,15].

Portugal offers a distinctive context for examining the potential of whey upcycling. Traditional cheese production is widely distributed across several regions, including Trás-os-Montes, generating significant volumes of whey that represent both an environmental burden and an opportunity for innovation [16,17,18]. These whey volumes are integrated into a broader value chain encompassing collection, processing, and potential reintegration into food and feed applications, illustrating the opportunities for sustainable utilization and development of functional ingredients within the Portuguese dairy sector [17,19]. Recent Portuguese studies have explored the reintegration of dairy by-products into food matrices as a strategy to enhance nutritional value while supporting circular economy principles [17,19,20], illustrating how whey can be repositioned from a residual stream to a functional resource within regional agro-food systems.

Building on this framework, this review examines the potential of whey-derived peptides as functional ingredients with cardiovascular and immunomodulatory significance. Specifically, this review aims to:

(i) describe the composition of cheese whey and associated dairy by-products, together with key management and environmental challenges;
(ii) summarize established and emerging upcycling pathways for whey proteins;
(iii) characterize the functional properties of whey-derived bioactive peptides;
(iv) critically evaluate evidence on cardiovascular effects across in vitro studies, animal models, and human clinical trials;
(v) synthesize current findings related to immunomodulatory actions; and
(vi) situate these developments within the Portuguese research and innovation landscape, highlighting opportunities for sustainable whey utilisation.

Given the heterogeneity of experimental models, outcome measures, and intervention designs, a narrative review approach is appropriate to integrate mechanistic insights, preclinical findings, and emerging human evidence. By linking peptide bioactivity with sustainability-oriented whey valorisation, this review seeks to clarify the translational potential of whey-derived peptides and to support the development of functional food ingredients relevant to cardiometabolic and immune health.

## 2. Cheese Whey: Composition, By-Products and Challenges

Cheese whey (CW) is the liquid by-product obtained after the precipitation of milk casein during the cheese-making process [21]. It represents one of the most abundant residual streams in the dairy industry. Approximately 80–90% of the milk volume used for cheese production is converted into whey, which retains around 50–55% of the original milk nutrients, highlighting its nutritional relevance despite being traditionally classified as a by-product [22,23]. Globally, cheese production reaches roughly 24 million tonnes per year, yielding an estimated 21–22 million tonnes of CW [23]. Its compositional richness and volume make CW central to both environmental management and valorization strategies in the dairy sector.

Two main types of cheese whey are distinguished based on coagulation: sweet whey and acid whey. Sweet whey is obtained through rennet-induced coagulation and is typical of most hard and semi-hard cheeses. In contrast, acid whey is produced by acid-induced coagulation or lactic acid bacteria fermentation, as in the production of cottage cheese or other acid-curdled cheeses [24]. These two types of whey differ in several physicochemical properties, including pH, protein, fat, lactose, and mineral content, which influence their technological behavior, protein recovery, and potential applications in food and functional ingredient development. Table 1 summarizes the main compositional differences between sweet and acid whey from bovine milk. Compared to sweet whey, acid whey usually contains slightly lower lactose but higher mineral content, which can influence downstream processing, protein recovery, and technological applications.

The protein fraction of cheese whey is mainly composed of β-lactoglobulin and α-lactalbumin, along with minor amounts of serum albumin, lactoferrin, and immunoglobulins, which confer high biological value and functional properties [24,25]. The main compositional differences among whey from different milk sources, including bovine, ovine, caprine, and second cheese whey (SCW), are summarized in Pires et al. (2021) [24]. Notably, ovine whey contains higher protein (1.6–1.8% *w*/*v*) and mineral content (1.0–1.8% w/v) than bovine whey (protein 0.7–0.9%, minerals 0.5–0.6%), while caprine whey shows intermediate values. These differences reflect natural variation between species, primarily due to physiological factors such as the animal’s metabolism and lactation stage, as well as environmental influences, including diet and season. Additionally, whey composition can be affected by milk processing and separation methods, which may further modulate protein and mineral concentrations.

In Mediterranean countries, including Portugal, a significant proportion of CW is traditionally further processed into whey cheeses such as ricotta, requesón, or requeijão. During these processes, sweet whey is heated and acidified to coagulate residual whey proteins and fine casein particles, forming a soft curd recovered as whey cheese. The remaining liquid, SCW or “sorelho,” is protein- and fat-poor but retains substantial lactose (3–5%) and soluble minerals, particularly sodium and calcium when brining is involved. SCW appears under different names in the literature (e.g., scotta, deproteinized whey, post-ricotta whey), and its composition varies according to milk origin and processing parameters [4,24,26]. Sciuto et al. (2025) [4] report that bovine SCW typically exhibits pH 6.0, protein 0.1–0.2%, lactose 4.8–5.0%, and salts 1.0–1.1%, whereas ovine SCW shows slightly higher pH (6.2–6.5) but lower protein (0.5%) and mineral (0.5%) content. Although often overlooked due to its limited protein content, SCW represents a relevant substrate for biotechnological valorisation, including fermentation-based routes and the generation of functional metabolites.

Given the scale of CW and SCW generation and their high biochemical oxygen demand and chemical oxygen demand, driven mainly by lactose and residual proteins, uncontrolled disposal of these streams can lead to severe oxygen depletion in receiving waters and contribute to eutrophication, making them up to one or two orders of magnitude more polluting than typical domestic wastewater [21,27,28]. This is of particular concern in regions with many small and artisanal dairies, where economic and logistical constraints can limit the adoption of advanced wastewater treatment technologies, and where SCW from whey-cheese production is still frequently discharged into municipal sewers or septic pits or applied to land without strict control [28,29,30].

In response to these challenges, the dairy industry has progressively adopted valorisation strategies including the production of whey powders, whey protein concentrates and isolates, lactose and demineralized whey powders, as well as the use of whey and permeate as ingredients in beverages, bakery and confectionery [17,24]. In parallel, fermentation-based and biorefinery approaches have been explored to convert CW and, increasingly, acid whey and SCW into single-cell proteins [31], bioethanol and biobutanol, organic acids, biogas, and bioplastics [6,32], thereby reframing these streams as valuable feedstocks rather than environmental burdens. Thus, the antimicrobial, antihypertensive, and prebiotic properties of this byproduct should be the subject of analysis for the valorization of its supply in the food industry and environmental awareness [33], which requires comprehensive studies and dedication to the innovation of viable processing techniques for obtaining these functional compounds in their separate, active form, suitable for inclusion in industrial production.

## 3. Upcycling Pathways for Whey Proteins

Whey was originally considered to be a low-value by-product of cheese production [3,12]. However, it is now widely recognized as a source of functional and bioactive compounds, especially proteins and peptides with high nutritional and physiological relevance [24]. This shift in perception reflects both increasing pressure to reduce food system waste and advances in processing technologies that enable the recovery and functionalization of whey constituents [12]. Despite this progress, it is estimated that approximately 40–50% of whey generated is still discarded globally [3,24].

In food applications, whey proteins and their derivatives are gaining attention due to their broad functional benefits, including gelation, foaming, thickening, emulsification, solubility, and thermal stability [34,35]. These properties enable their incorporation into a wide range of formulations, where they act not only as nutritional components but also as structural agents that influence texture, stability, and overall product performance [34]. Commercially available whey protein products include whey powder (8–12% protein and over 70% lactose), whey protein concentrate (WPC, protein concentration 30–89%), whey protein isolate (WPI, protein concentration 90–95%), whey protein hydrolysate (WPH, protein concentration < 80%), and isolated whey proteins [24,36]. Their technological versatility has supported the development of new food systems and facilitated their incorporation into a wide range of product categories, including dairy-based items, beverages, sports nutrition formulations, desserts, infant formulas, dietetic products, ready-to-eat meals, bakery goods, and confectionery [3].

Recent advances in processing and formulation technologies have expanded whey utilization beyond conventional ingredients toward higher-value and function-oriented products. Strategies such as fermentation, enzymatic hydrolysis, encapsulation, and emerging digital food technologies have facilitated the development of innovative matrices that combine nutritional enhancement with functional bioactivity. Representative examples of contemporary whey-based food formulations are summarized in Table 2, illustrating how whey or its protein fractions are integrated into fermented dairy products, beverages, and bakery items to improve protein content, antioxidant capacity, sensory attributes, and overall sustainability.

**Table 2:** Recent innovations and formulations of whey-derived food products.

Product	Whey Source	Main Goals/Achievements	Whey Effect on Product Properties	References
Whey-based yogurt incorporating cushuro (*Nostoc sphaericum*) and mango jam	Liquid whey (upcycled)	- Promoting sustainability and functionality in dairy processing - 3D printing in the development of new food matrices - Ideal Profile (IP) for consumers using the Check All That Apply (CATA) method	Provides proteins and lactose; supports fermentation and viscosity; contributes to sweetness, aroma and creaminess	[37]
Whey protein yogurt enriched with nanoencapsulated yellow *Capsicum annuum* extract	Whey protein isolate	- Functional dairy product with enhanced nutritional and sensory qualities - Nanoencapsulation of bioactive components to improve nutritional profile, product stability, and shelf-life	Increases protein content; improves viscosity, stability and overall acceptability	[38]
Biscuits using whey protein and *Ocimum gratissimum*	Replacing whole wheat flour with WPC	Replacing whole wheat flour with WPC, improving nutritional parameters, and its antioxidant activity	Increases protein, fat, minerals and digestibility; modifies volume, density and spread; supports acceptable sensory quality	[39]
Fermented whey-based sports beverage fortified with *Spirulina platensis*	Ricotta cheese whey (upcycled)	- Improve textural quality, sensory characteristics, and improving rheological properties - Improve nutritional value and nutraceutical properties by enhancing antioxidants	Served as fermentation substrate; supported probiotic viability; provided nutrients and functional base for beverage	[40]
Rye bread fortified with whey	Milk whey replaced water as the formulation liquid component (upcycled)	- Development of functional, nutritional, and sustainable foods - Consumer acceptability evaluation	Increased protein and minerals; reduced moisture; modified crust color and texture; maintained microbial stability; high consumer acceptance	[17]
Innovative whey cheese (Lor)	Kashar cheese whey (upcycled)	- Development of fermented whey cheeses with improved functional properties - Increase antioxidant activity, while also improving the sensory characteristics	Provided whey proteins and nutrients; enabled whey cheese production and matrix formation	[41]
Whey cheese (*Requeijão*) with Kefir or probiotics	Bovine cheese whey concentrated by ultrafiltration	- Efficient production of bovine whey cheeses - Substantial reduction of energy costs - Improving nutritional and sensory characteristics, alongside the potential for the extension of whey cheese shelf-life	Provided whey proteins and nutrients; enabled whey cheese production and matrix formation	[42]

The examples summarised in Table 1 highlight a clear trend toward the incorporation of whey into food systems designed to address current consumer and industry demands, including nutritional fortification, clean-label reformulation, and waste reduction. One notable trend is the integration of whey into fermented and dairy-based matrices, as seen in the development of fortified yogurts and functional beverages, as they combine biological transformation with improved digestibility and bioaccessibility of proteins and peptides. These approaches exploit the nutritional quality of whey proteins while enabling the incorporation of bioactive compounds, either through natural sources or through technological interventions. Such strategies improve protein content, enhance antioxidant capacity, and extend shelf-life, underscoring the potential of whey to support the creation of nutritionally enriched, consumer-oriented products.

Another relevant pathway involves the substitution of traditional ingredients with whey protein concentrates or liquid whey in bakery applications. Formulations such as whey-enriched biscuits and whey-fortified rye bread highlight the capacity of whey to enhance protein levels, influence textural attributes, and contribute to the development of more sustainable food systems by partially replacing staple ingredients such as wheat flour or water. These examples show how whey utilisation can be aligned with contemporary goals in product reformulation, including nutritional improvement, waste reduction, and diversification of functional foods.

Whey proteins can be utilised to produce bioactive peptides and modified protein fractions with functional properties. The production of whey protein hydrolysates enriched in low-molecular-weight peptides has intensified in recent years, supported by advances in enzymatic processing and analytical characterization. These hydrolysates can be incorporated into foods, beverages, and specialized nutritional products, offering a bridge between technological valorization and health-oriented functionality. Chen et al. [43] conducted a study in which whey protein hydrolysates were incorporated into an existing infant formula, and the peptide profiles were compared before and after simulated digestion. The authors identified 126 bioactive peptides with immune, antimicrobial, sleep-regulating, and neurological potential, supporting the feasibility of enhancing infant formula with whey protein hydrolysates to increase its bioactive potential.

Whey proteins also serve as versatile building blocks for functional delivery systems due to their intrinsic physicochemical properties. Depending on processing conditions, they can be structured into macro-(films, coatings, beads), micro-(microgels, microencapsulation systems, microspheres), or nano-(nanohydrogels, nanofibrils, nanoparticles, nanoemulsions)scale assemblies capable of encapsulating and protecting bioactive compounds while enabling controlled release within food matrices [34,44]. These protein-based architectures present considerable potential within the food sector, particularly as advanced delivery systems capable of encapsulating and enabling the controlled release of functional compounds, flavour molecules, and essential nutrients [44]. For example, Goudali et al. [45] developed an innovative WPI-based biopolymer film with biodegradability and improved food preservation capabilities, offering a promising alternative to conventional plastics. In another work, Feng et al. [46] studied the release and bioaccessibility of β-carotene from 3D printed delivery systems based on emulsion gels stabilized by whey protein (WPI)/polysaccharide.

Overall, whey protein upcycling encompasses a continuum of strategies ranging from conventional ingredient recovery to advanced peptide generation and functional delivery systems. Within the scope of this review, particular emphasis is placed on upcycling pathways that facilitate the production and effective application of whey-derived bioactive peptides, as these represent the most direct link between sustainability-driven valorisation and cardiovascular and immunomodulatory health outcomes. This focus provides the foundation for the subsequent sections, which critically examine the biological activity and strength of evidence supporting whey-derived peptides as functional food ingredients.

## 4. Bioactive Peptides and Functional Properties of Whey Proteins

Whey proteins, accounting for roughly 18–20% of total milk nitrogen, are key precursors of bioactive peptides generated during gastrointestinal digestion, controlled enzymatic hydrolysis, or microbial fermentation [12,15,47]. The major proteins include β-lactoglobulin (50–55% of whey proteins), α-lactalbumin (20–25%), bovine serum albumin (5–10%), immunoglobulins (10–15%), lactoferrin (1–2%), and glycomacropeptide (20–25%) [12,48], each yielding distinct peptide profiles upon proteolysis. These peptides are typically short sequences (2–20 amino acids) in which hydrophobic residues, cationic side chains, and proline-rich motifs confer antioxidant, antimicrobial, antihypertensive, and immunomodulatory properties [12,35].

As summarized in Table 3, the main functional classes of whey-derived bioactive peptides can be classified according to their precursor proteins, representative sequences, and mechanisms of action. β-Lactoglobulin is the primary source of ACE-inhibitory peptides such as β-LG f(78–91) (IPAVFKIDALNENK), which competitively binds angiotensin-converting enzyme to reduce angiotensin II formation, thereby promoting vasodilation and blood pressure reduction. This class also includes sequences from α-lactalbumin and lactoferrin that contribute to cardiovascular protection through similar mechanisms [12,15,49,50].

Antioxidant peptides, predominantly from β-lactoglobulin and α-lactalbumin (e.g., β-LG f(18–29), α-LA f(53–61)), scavenge reactive oxygen species, chelate transition metals, and activate endogenous defenses like the Nrf2 (Nuclear Factor Erythroid 2-Related Factor 2) pathway, protecting vascular endothelium and lipoproteins from oxidative damage [51,52,53,54].

Whey proteins are also recognized as a source of antimicrobial and immunomodulatory peptides with potential relevance to gut and systemic immunity. Antimicrobial peptides such as lactoferricin (LF f(17–41)) from lactoferrin and β-lactoglobulin-derived sequences disrupt microbial membranes through cationic interactions and pore formation, supporting pathogen control and gut microbiota balance [15,55,56]. Immunomodulatory peptides from α-lactalbumin, lactoferrin, and immunoglobulins (e.g., α-LA f(16–26)) modulate cytokine profiles by downregulating pro-inflammatory mediators (TNF-α, IL-6) and enhancing anti-inflammatory signals (IL-10), while promoting mucosal immunity through IgA production and NF-κB signaling [57,58,59]. These mechanisms provide a biological rationale for the immunomodulatory effects examined in Section 6.

Table 3 illustrates how these mechanisms translate into physiological benefits: ACE inhibition supports cardiovascular function through blood pressure regulation; antioxidants limit oxidative damage to vascular structures; antimicrobials contribute to infection control and microbiota homeostasis; and immunomodulators promote balanced immune responses. 

**Table 3:** Functional classes of whey-derived bioactive peptides: precursors, representative sequences, mechanisms, and health relevance.

Class	Main Precursors	Representative Peptide(s)	Principal Mechanism of Action	Health Relevance	References
ACE-inhibitory	β-LG, α-LA, BSA, LF	β-LG f(78–91)	Competitive ACE binding reduces angiotensin II formation and promotes vasodilation	Blood pressure reduction, vascular protection	[49,50]
Antioxidant	β-LG, α-LA	β-LG f(18–29)	Scavenges reactive oxygen species, chelates metals, and activates the Nrf2 pathway	Endothelial protection, reduced oxidative stress	[12,15,54]
Antimicrobial	Lactoferrin, β-LG	LFcin f(17–41)	Disrupts microbial membranes through cationic interactions and pore formation	Pathogen control, gut health	[15,60,61]
Immunomodulatory	α-LA, LF, IgG, GMP	α-LA f(16–26)	Modulates cytokines (reduced TNF-α/IL-6, increased IL-10), enhances IgA production	Inflammation regulation, mucosal immunity	[12,15,54,57]

Many whey-derived peptides exhibit multifunctional activity, and complex hydrolysates from whey protein concentrates often demonstrate synergistic antihypertensive, antioxidant, and anti-inflammatory effects [12,33,62].

Despite this promising functional profile, several technological and translational challenges remain. These challenges include controlling hydrolysis conditions to optimize the release of bioactive peptides while minimizing bitterness, ensuring peptide stability during gastrointestinal transit, achieving reproducible bioactivity across production batches, and complying with regulatory requirements for substantiated health claims. Such limitations are particularly relevant when upcycling complex streams such as acid whey and second cheese whey, highlighting the need for integrated approaches that combine process optimization, bioactivity assessment, and food system applicability. Addressing these challenges is essential for advancing whey-derived bioactive peptides from experimental systems toward validated functional food ingredients [63,64].

In addition to the well-characterized antihypertensive, antioxidant, antimicrobial, and immunomodulatory activities summarized in Table 3, whey-derived peptides exhibit multifunctional properties, including anti-inflammatory and antithrombotic effects, which contribute to vascular homeostasis and systemic health [12,49,50,51]. These properties, together with the potential for synergistic interactions among peptide sequences, highlight the broad functional repertoire of whey proteins and their suitability as bioactive ingredients in functional foods, with the primary cardiovascular and immunomodulatory effects explored in detail in Section 5 and Section 6.

## 5. Cardiovascular Effects of Whey-Derived Peptides

**Figure 1:**
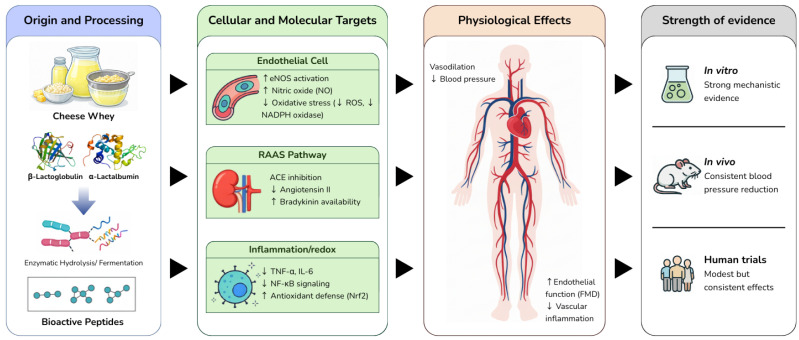
Mechanistic pathways linking whey-derived peptides to cardiovascular effects across biological levels. Schematic representation of the origin, molecular targets, physiological outcomes, and evidence strength of whey-derived bioactive peptides. **Panel 1** shows peptide origin from cheese whey, including milk processing and enzymatic hydrolysis or fermentation of whey proteins (β-lactoglobulin, α-lactalbumin). **Panel 2** highlights key cellular targets, including endothelial eNOS activation and NO production, RAAS inhibition via ACE suppression, and modulation of inflammation and redox signaling (TNF-α, IL-6, NF-κB, Nrf2). **Panel 3** depicts physiological outcomes, such as vasodilation, reduced blood pressure, improved endothelial function (FMD), and decreased vascular inflammation. **Panel 4** summarizes evidence strength across in vitro and clinical studies.

Cardiovascular diseases (CVDs) remain the leading cause of morbidity and mortality worldwide, with hypertension and endothelial dysfunction recognized as major modifiable risk factors [65,66]. Dietary interventions targeting vascular function have gained attention, particularly bioactive peptides derived from food proteins. Whey proteins, abundant in dairy by-products, represent a promising source of such peptides due to their well-characterized amino acid composition and capacity to release bioactive sequences during enzymatic hydrolysis or gastrointestinal digestion [10,67]. At a molecular level, whey-derived peptides have been reported to exert antihypertensive effects through angiotensin-converting enzyme (ACE) inhibition, modulation of nitric oxide (NO) bioavailability, antioxidant activity, and anti-inflammatory actions [49,68]. An integrated overview of the mechanistic pathways and the relative strength of evidence supporting the cardiovascular effects of whey-derived peptides across in vitro, in vivo, and human studies is provided in Figure 1.

### 5.1. In Vitro: ACE Inhibition, Nitric Oxide Modulation, and Antioxidant Activity

The antihypertensive potential of whey peptides has been extensively characterized at the molecular level, as exemplified by Chamata et al. [49], who employed molecular docking to predict the binding affinity of specific whey-derived peptides to ACE, revealing sequences with strong inhibitory potential [47]. Similarly, Czelej et al. [47] performed enzymatic hydrolysis of whey proteins, followed by peptide profiling, identifying several short-chain sequences capable of potent ACE inhibition in vitro [69]. These findings reveal the mechanistic basis for the antihypertensive properties of whey peptides and provide a rational framework for subsequent in vivo and clinical investigations.

Beyond ACE inhibition, additional mechanisms have been proposed, such as whey-derived peptides that exhibit antioxidant activity by scavenging reactive oxygen species and enhancing endothelial NO production, contributing to vasodilation [49,68]. Furthermore, emerging evidence suggests that certain peptides may interact with the gut microbiota, indirectly modulating vascular tone and blood pressure through microbial metabolite production, as observed in rodent models [70]. Taken together, these molecular and cellular studies support the hypothesis that whey peptides can influence multiple pathways relevant to cardiovascular health.

### 5.2. In Vivo Evidence: Blood Pressure and Vascular Function in Animal Models

Animal studies provide further insights into the physiological relevance of whey peptides, as demonstrated by a recent study using hypertensive rat models, which demonstrated that whey protein hydrolysates significantly reduced systolic and diastolic blood pressure, with concomitant improvements in vascular reactivity [70]. The effects were partially attributed to modulation of the gut microbiome and the generation of bioactive peptide fragments, reinforcing the translational potential of these compounds. These observations are consistent with in vitro evidence and underscore the importance of peptide-specific activity in mediating cardiovascular outcomes.

In spontaneously hypertensive rat (SHR) models, daily oral administration of whey hydrolysates or enriched peptide fractions has repeatedly been shown to lower systolic and diastolic blood pressure over treatment durations ranging from several days to multiple weeks. Typical reductions range from 10 to 25 mmHg depending on dose, peptide composition and baseline severity of hypertension and these blood pressure lowering effects are frequently accompanied by significant modulation of the renin–angiotensin–aldosterone system (RAAS), including reduced plasma angiotensin II levels, decreased ACE activity in renal and vascular tissues, and improved expression of vasodilatory mediators [71,72,73]. Besides that, improvements in vascular function, with isolated vessel assays showing enhanced acetylcholine-induced vasodilation and reduced contractile responses to vasoconstrictors, suggest restored endothelial responsiveness and improved smooth muscle signaling [74,75,76]. Collectively, these data support a composite mechanism in which whey-derived peptides moderate RAAS activity, mitigate oxidative stress and inflammation, and restore endothelial signalling pathways that are typically impaired in hypertension.

### 5.3. Human Evidence: Blood Pressure, Endothelial Function, and Lipid Profile

Clinical research on whey-derived peptides has focused on blood pressure regulation and endothelial function, as illustrated by the Whey2Go randomized controlled trial reported by Fekete et al. [66], which investigated adults with prehypertension or mild hypertension over an 8-week intervention period. Whey protein supplementation led to a significant reduction in both systolic and diastolic blood pressure, accompanied by improvements in flow-mediated dilation (FMD) and lipid biomarkers [66]. Acute effects have also been demonstrated through NOP-47 peptide extract administration, which enhanced endothelial function in adults at cardiovascular risk in a randomized crossover trial, indicating that specific whey peptides can rapidly influence vascular tone [65]. Additional randomized controlled trials reporting similar outcomes are summarized in Table 4, highlighting differences in population characteristics, intervention type, dose, and duration.

Narrative reviews further support these outcomes, as reported by [10], which summarize multiple human interventions and conclude that whey protein and its hydrolysates improve endothelial function and may contribute to modest blood pressure reductions. However, heterogeneity in study design and peptide composition limits the ability to draw definitive conclusions. Major sources of variability include differences in whey source (intact protein versus hydrolysate), degree and method of enzymatic hydrolysis, peptide characterization, administered dose, intervention length (acute versus chronic), and baseline health status of participants (normotensive, prehypertensive, or clinically compromised populations). In several studies, incomplete characterization of peptide fractions further limits mechanistic interpretation.

Meta-analyses corroborate these observations, with a systematic review and meta-analysis of human trials, as described in [70], confirming a positive effect of whey protein on FMD and vascular health, while Prokopidis et al. [80] highlighted improvements in blood pressure and vascular function attributable to bioactive whey peptides. Nevertheless, reported effect sizes are generally small and appear to be influenced by baseline cardiovascular risk and intervention characteristics. Overall, current evidence supports a beneficial role of whey-derived peptides in vascular function, although greater standardization in peptide profiling and trial design is needed to strengthen causal inference.

### 5.4. Promising Peptide Candidates and Mechanistic Integration

Specific peptide sequences derived from β-lactoglobulin, α-lactalbumin, and lactoferrin have emerged as particularly promising in modulating cardiovascular endpoints [67,68]. Lactokinins, for example, exhibit strong ACE-inhibitory activity and improve endothelial function via NO-mediated vasodilation, with antioxidant and anti-inflammatory effects that complement their antihypertensive action, creating a multifaceted cardioprotective profile [47,49]. The interplay between the direct bioactivity of peptides and their modulation of gut microbiota may contribute to improved vascular outcomes, but robust clinical investigations are needed to verify these pathways in humans.

Mechanistically, the cardiovascular actions of these peptides appear multifactorial. ACE inhibition remains a central pathway, as reducing Ang II formation and slowing bradykinin degradation directly promotes vasodilation and alleviates vascular constriction. However, the accumulating evidence indicates that their effects extend beyond RAAS modulation alone. Many whey-derived peptides exhibit antioxidant activity capable of reducing NO inactivation and preserving endothelial signaling [14,81,82]. Some peptides also influence intracellular kinase pathways associated with eNOS activation, thereby increasing NO production [83,84]. In addition, anti-inflammatory effects have been documented, suggesting that these peptides may reduce endothelial dysfunction by dampening inflammatory signaling cascades implicated in hypertension and vascular disease [10,57]. This constellation of interconnected mechanisms enhances the biological plausibility of whey peptides as functional cardioprotective agents and supports their classification as multi-target bioactives rather than isolated enzyme inhibitors.

### 5.5. Evidence Quality, Dose Considerations, and Translational Limitations

Despite encouraging findings, several limitations warrant consideration, as human trials exhibit considerable heterogeneity in terms of whey source, hydrolysis method, peptide characterization, and intervention duration [10,65,66,80]. Moreover, translation from animal models to human physiology remains uncertain, particularly regarding dose equivalence and bioavailability. Standardization of whey hydrolysates and precise identification of bioactive sequences are, therefore, essential for the development of functional foods or nutraceuticals targeting cardiovascular health, while long-term clinical trials are required to confirm sustained benefits and safety.

## 6. Immunomodulatory Effects of Whey-Derived Peptides

**Figure 2:**
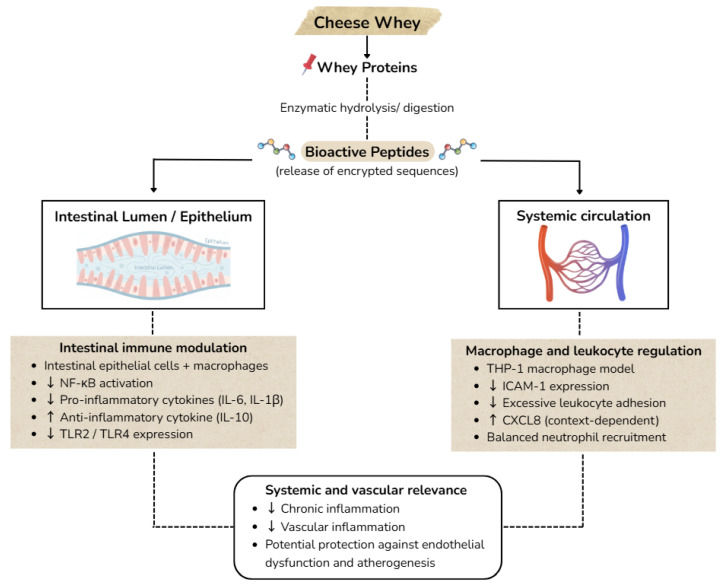
Immunomodulatory pathways of whey-derived peptides at the intestinal and systemic level.

Bioactive peptides derived from whey proteins have attracted considerable interest due to their health-promoting effects, particularly their immunomodulatory potential. Most studies have focused on whey protein isolate, while peptides generated from cheese whey or second cheese whey have been less extensively investigated. In the gastrointestinal tract, these peptides are released by digestive and pancreatic enzymes and can either enter the bloodstream after absorption or act locally on luminal tissues [57,85,86,87]. The main immunomodulatory mechanisms of whey-derived peptides involve modulation of both innate and inflammatory immune responses, as schematically summarized in Figure 2.

The degree of digestion strongly influences the immunomodulatory activity, with more extensively digested peptides generally exhibiting stronger anti-inflammatory effects on immune cells. These peptides can suppress the NF-κB pathway through PPARγ-dependent mechanisms, reduce pro-inflammatory cytokines such as IL-6 and IL-1β, and modulate Toll-like receptor expression following inflammatory stimuli. In this way, NF-κB activation is attenuated, leading to a more controlled inflammatory response and promoting an immunoregulatory phenotype. For example, TLR2 expression decreases upon exposure to whey peptides regardless of the animal of origin, whereas TLR4 downregulation appears more pronounced with cheese-derived peptides. Both bovine and ovine whey proteins have been reported to reduce activation of inflammatory cascades [57,87].

Whey-derived peptides also influence immune cell recruitment and chronic inflammation. CXCL8 transcription increases in response to these peptides when immune cells are stimulated by inflammatory factors, facilitating neutrophil recruitment during the acute phase of inflammation. At the same time, IL-10 expression is elevated in LPS-activated THP-1 macrophages exposed to mixed-origin whey peptides, providing anti-inflammatory regulation that may be particularly beneficial in chronic intestinal inflammation. In addition, ICAM1 mRNA expression is reduced, especially with bovine or mixed-origin peptides, which limits leukocyte adhesion and vascular inflammation and may contribute to protection against atherosclerotic lesion formation [57,88,89,90,91].

Importantly, the immunomodulatory efficacy of whey peptides depends on peptide source, degree of hydrolysis, concentration, and immune context. TNF expression and macrophage gene profiles can be differentially affected depending on peptide digestion, and high concentrations of fully digested peptides may sometimes produce inverse effects on inflammatory responses [57,87,92]. Overall, these findings indicate that whey-derived peptides can modulate inflammation and immune function, but the response is highly context-dependent, underscoring the need for further studies to clarify these interactions.

## 7. Portuguese Perspective: Research, Industry, and Opportunities

**Figure 3:**
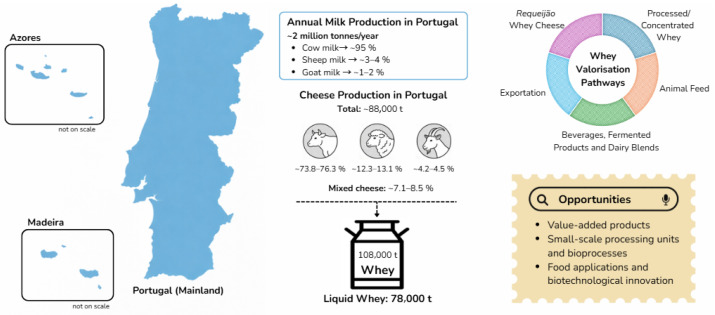
Milk production and whey valorisation in Portugal. The figure illustrates the production (tonnes/year) of raw milk and cheese manufacturing by animal species and associated whey generation in Portugal. The data is based on statistics records kept by the Portuguese Institute of National Statistics (INE) during the 2022–2024 period [93]. The schematic identifies current valorisation pathways and future opportunities for industrial whey valorisation, according to the most recent studies conducted over five years.

In Portugal, whey valorisation has historically been closely linked to traditional dairy practices, particularly through the production of whey-derived cheeses such as Requeijão, as previously mentioned in Section 2. This product is traditionally manufactured from ovine, caprine, bovine, or buffalo cheese whey and represents one of the most established routes for whey utilisation within the national dairy sector [42]. Despite its cultural and gastronomic relevance, the conventional yield of Requeijão remains relatively low, typically around 6% of the initial whey volume [42], which limits its effectiveness as a large-scale valorisation strategy for the total whey generated by the industry. In addition, traditional whey valorisation through Requeijão production is constrained by high energy consumption and short shelf life, further limiting its economic and environmental sustainability as a large-scale valorisation pathway. An overview of the Portuguese dairy landscape, including major whey-producing regions, research activity, and valorisation pathways, is presented in Figure 3.

Beyond traditional processing, the Portuguese dairy sector still exhibits a relatively modest level of industrial whey valorisation. Available data on the fate of cheese whey in Portugal remain limited and largely based on estimates reported in the literature. Existing studies suggest that approximately half of the bovine whey generated annually—estimated at around 300,000 tonnes—is processed into commercial products, resulting in roughly 13,000 tonnes of whey powder (12% protein) [24]. The majority of the remaining whey is diverted to animal feeding practices, reflecting the limited exploitation of its potential for higher-value applications [24,94]. In a study conducted in the Baixo Alentejo region, surveys were carried out in 47 cheese factories across 13 municipalities to characterize the daily production and management of goat cheese whey and sheep second whey cheese. With a 60% response rate, the total daily cheese whey production was estimated at 21,237 L. Approximately 57% of dairies did not reuse whey, disposing of it in pits or into the public sewage system, 36% supplied it to livestock producers as feed, and 7% were unable to provide this information [95]. These practices highlight the environmental burden associated with inadequate whey management and reinforce the need for waste reduction and circular valorisation strategies within the Portuguese dairy sector.

From a research perspective, there has been interest in exploring alternative and higher-value applications for whey and whey proteins in Portugal. In a recent study, Moura-Alves et al. [17] developed a rye bread using liquid whey as a replacement for water. This approach resulted in a protein content that was approximately 15% higher than that of the control formulation, which utilised water as the primary liquid component. Furthermore, the potassium and fibre content of whey formulations was enhanced. The authors demonstrated the feasibility of incorporating whey into bread formulations as a strategy to enhance nutritional value and promote ingredient circularity. Other studies have reported the incorporation of whey into different food formulations, including dairy-based beverage products with prebiotic properties and systems fermented with probiotic bacteria, further illustrating the adaptability of whey as a functional ingredient across diverse product concepts [96,97,98]. Henriques et al. [20] studied the production of acid gels that were produced by lactic acid fermentation (yogurt-type gels) or acidification (dessert-type gels) using liquid whey.

Whey proteins have also been explored for their technological functionality within food systems, particularly in the development of edible and active materials. Robalo et al. [99] assessed the performance of an edible active film based on whey protein concentrate enriched with green tea extract for the preservation of fresh cheeses. The results demonstrated effective inhibition of *E. coli* growth and a significant delay in lipid oxidation, highlighting the potential of this approach to extend the shelf life of fresh cheese. Similarly, Castro et al. [100] reported the effectiveness of an edible WPC film incorporated with green tea extract in retarding lipid oxidation in fresh salmon, further supporting the applicability of whey protein films as active packaging materials for food preservation.

Despite scientific evidence supporting the valorisation of whey and its derivatives, there remains considerable scope for improvement in Portugal. The industry is dominated by a large number of micro, small, and medium-sized cheese producers, which often lack the technological capacity, infrastructure, or economic scale required for systematic whey processing and valorisation. In practice, small and medium-sized enterprises account for approximately 40–50% of cow cheese production and bovine whey generation [101]. For many of these operators, whey continues to be perceived primarily as a management burden rather than a strategic resource, as its handling, treatment, or disposal entails additional operational costs with limited direct economic return. As a result, whey is frequently managed at a local level, most commonly through direct use in animal feeding, without the integration into organised value chains. Beyond scale constraints, the sector faces persistent structural limitations, including low levels of technological innovation, limited diversification into higher-value whey-based products, dependence on imported processing inputs, and insufficient integration of energy-efficient and circular approaches to by-product management [101]. These conditions help explain the persistent gap between scientific potential and industrial implementation, highlighting the need for coordinated policy instruments, shared processing infrastructure, and collaborative value-chain models capable of enabling the practical adoption of higher-value whey utilisation strategies within the Portuguese dairy sector.

In this context, the *Lacties* project [101] has sought to promote the sustainable and circular valorisation of whey and dairy by-products, enhance technological innovation among small and medium-sized producers, and foster the development of eco-efficient and economically viable value chains. Opportunities for improvement may include the development of whey-based food formulations, ingredients, and beverages, the adoption of innovative processing technologies to create higher-value products, and the establishment of shared regional processing hubs to achieve economies of scale. Further potential lies in enhancing eco-efficiency and energy optimisation in production, developing local resources and co-products to replace imports and strengthen value chains, and implementing systematic mechanisms to reduce environmental impact.

## 8. Evidence Synthesis and Research Gaps

This review highlights a paradigm shift in the perception of whey and its by-products, including cheese whey, which are increasingly transitioning from being regarded as polluting residues to valuable resources within sustainable food systems. Rather than representing an environmental burden alone, these by-products are now recognized for their potential to deliver functional value to foods and to contribute to cardiovascular health and immunomodulation, largely due to the presence of high-value bioactive peptides.

Evidence from in vitro and in vivo animal studies shows a convergence toward the bioactivity of whey-derived peptides, particularly lactokinins, and other ACE inhibitor peptides derived mainly from α-lactalbumin and β-lactoglobulin, as discussed in Section 4 and Section 5. Through ACE inhibition and the consequent reduction in angiotensin II formation, these peptides attenuate vasoconstriction and support blood pressure regulation. In parallel, their ability to modulate NO signalling and oxidative balance contributes to reduced lipid peroxidation, enhanced antioxidant defenses, and preservation of endothelial integrity. Collectively, these mechanisms underpin improvements in vascular function and stabilization of endothelial signaling pathways (Figure 2). At the immunomodulatory level, whey-derived peptides demonstrate the capacity to suppress NF-κB-dependent inflammatory pathways and to regulate pro-inflammatory cytokine production, supporting a shift toward an immunoregulatory phenotype associated with reduced vascular inflammation and improved homeostasis.

The multifunctional nature of these peptides acting across interconnected physiological pathways suggests their relevance for human health (Section 5 and Section 6). However, while clinical trials indicate modest cardiometabolic benefits, particularly among individuals at cardiovascular risk, the evidence remains limited by substantial methodological heterogeneity of the studies. Most human interventions evaluate whole whey proteins or complex hydrolysates rather than defined peptide preparations, preventing definitive attribution of observed effects to specific bioactive sequences and limiting causal interpretation.

Critical research gaps remain to be addressed. These include the need for standardized peptide production and characterization, clearer definition of effective concentrations, degrees of hydrolysis, and delivery matrices, as well as a marked scarcity of human pharmacokinetic data. Understanding peptide stability, absorption, metabolism, and target tissue exposure following oral intake is essential to establish effective and safe dosing strategies and appropriate intervention durations. In addition, longer-term human studies are required to evaluate the persistence and clinical relevance of observed effects over time.

From a sustainability and industrial perspective, despite growing efforts to optimize whey valorisation strategies, the potential for recovering bioactive peptides from cheese whey remains underexploited. Given the high availability of whey and its relevance to environmental pollution, integrating peptide-oriented upcycling approaches into scalable and economically viable processing models represents a key opportunity to align functional food innovation with circular economy principles, as observed in Section 3.

## 9. Conclusions and Future Perspectives

Advances in proteomics, peptide profiling, and molecular analysis have significantly contributed to reshaping the perception of cheese whey, demonstrating how a by-product traditionally regarded as an environmental pollutant can be transformed into a valuable source of functional ingredients. The growing body of evidence reviewed herein highlights the potential of whey-derived peptides to modulate key physiological pathways involved in cardiovascular regulation, including blood pressure control, endothelial integrity, oxidative stress balance, and inflammatory responses. This dual role—combining environmental mitigation with functional health promotion—positions upcycling as a compelling avenue for innovation within sustainable nutrition and functional food development, while actively supporting circular economy principles.

Looking forward, a central research priority will be the identification and validation of specific whey-derived peptide sequences with demonstrated efficacy and bioavailability in humans. This will require integrated research strategies combining controlled peptide production, advanced bioanalytical characterization, and robust pharmacokinetic assessments. In parallel, longer-term and adequately powered clinical intervention studies are needed, incorporating relevant mechanistic and functional biomarkers—such as angiotensin-converting enzyme activity, markers of endothelial function, oxidative stress, and inflammation—to establish clear dose–response relationships and provide clinical validation of observed effects. These steps are essential to move from promising experimental evidence toward credible functional applications suitable for incorporation into food products.

Beyond scientific challenges, the successful translation of whey-derived bioactive peptides into sustainable food systems will depend on coordinated efforts across academia, industry, and policy frameworks. Strengthening collaboration between these sectors is crucial to foster technological innovation, facilitate the adoption of scalable peptide recovery processes, and create regulatory and economic conditions that support the valorisation of functional ingredients. Such coordination is particularly relevant for countries like Portugal, where the dairy sector is dominated by small and medium-sized traditional cheese producers and where cardiovascular diseases represent a major public health burden. In this context, whey protein upcycling offers a unique opportunity to simultaneously reduce environmental impact, enhance regional economic resilience, and contribute to improved population health through the development of evidence-based functional foods.

## Figures and Tables

**Table 1:** Physicochemical composition of sweet and acid bovine cheese whey, including protein, fat, lactose, minerals, and pH. Data adapted from [24].

Parameter	Sweet Whey (Rennet Coagulation)	Acid Whey (Acid/Fermented Coagulation)
pH	6–7	4.5–5.8
Protein (g/L)	6–10	6–8
Fat (g/L)	5–6	5–6
Lactose (g/L)	46–52	44–46
Minerals (g/L)	2.5–4	4.3–7.2

**Table 4:** Overview of key randomized controlled trials evaluating the cardiovascular effects of whey protein and whey-derived peptides in human populations.

Population	Study Design	Intervention	Peptide Characterization	Dose and Duration	Primary Outcome	Effect Size	Reference
Adults with prehypertension and mild hypertension (*n =* 42)	Randomized, double-blind, 3-way crossover	Whey protein isolate	Intact whey protein; specific bioactive peptides not individually quantified	56 g/day; 8 weeks	24-h SBP, DBP, FMD, lipid biomarkers	−4.0 mmHg SBP; −2.8 mmHg DBP; +1.3% FMD (*p* < 0.05)	[66]
Overweight and obese adults with prehypertension (*n =* 65)	Randomized, controlled, parallel	Whey protein	Peptide profile not specified; intact whey	30 g/day; 12 weeks	SBP, DBP, endothelial function	−3.9 mmHg SBP in overweight subgroup (*p* < 0.05); improved FMD	[77]
Healthy adults at cardiovascular risk (*n =* 20)	Randomized, double-blind, crossover	Whey-derived peptide fraction (NOP-47)	Characterized peptide extract (NOP-47)	5 g (single administration); acute	FMD	+1.5–2.0% FMD increase vs placebo (*p* < 0.05)	[14]
Adults with elevated blood pressure (*n =* 30)	Randomized, crossover	Whey protein	Intact whey protein; no detailed peptide profiling	28 g (single meal); Acute postprandial	Postprandial SBP; FMD	−3 to −4 mmHg postprandial SBP; improved FMD (*p* < 0.05)	[78]
Patients with stable chronic heart failure (NYHA I–II) (*n =* 25)	Randomized, placebo-controlled	Whey protein supplementation	Intact whey; no peptide characterization	30 g/day; 12 weeks	Microvascular endothelial function	Significant improvement in endothelium-dependent vasodilation (*p* < 0.05)	[79]

SBP, systolic blood pressure; DBP, diastolic blood pressure; FMD, flow-mediated dilation; NYHA, New York Heart Association. Acute indicates single-dose or short-term postprandial assessment. Effect sizes are reported as described in the original publications. When detailed peptide profiling was not provided in the original study, this is indicated accordingly.

## Data Availability

No new data were created or analysed in this study. Data sharing does not apply to this article.

## Abbreviations

The following abbreviations are used in this manuscript:

3D	Three-Dimensional
ACE	Angiotensin-Converting Enzyme
α-LA	α-Lactalbumin
β-LG	β-Lactoglobulin
CW	Cheese Whey
CXCL8	C-X-C Motif Chemokine Ligand 8 (IL-8)
CVDs	Cardiovascular Diseases
eNOS	Endothelial Nitric Oxide Synthase
FMD	Flow-Mediated Dilation
ICAM-1	Intercellular Adhesion Molecule 1
Ig	Immunoglobulin
IL	Interleukin
LF	Lactoferricin
mRNA	Messenger RNA
NF-κB	Nuclear Factor Kappa-Light-Chain-Enhancer of Activated B Cells
NO	Nitric Oxide
Nrf2	Nuclear Factor Erythroid 2-Related Factor 2
PPARγ	Peroxisome Proliferator-Activated Receptor Gamma
RAAS	Renin–Angiotensin–Aldosterone System
ROS	Reactive Oxygen Species
SCW	Second Cheese Whey
SHR	Spontaneously Hypertensive Rat
THP-1	Human Monocytic Leukemia Cell Line
TLR	Toll-Like Receptor
TNF	Tumor Necrosis Factor
WPC	Whey Protein Concentrate
WPH	Whey Protein Hydrolysate
WPI	Whey Protein Isolate
